# Homeless people as target population in the scientific investigation

**DOI:** 10.3109/03009734.2011.638405

**Published:** 2012-02-15

**Authors:** Margit Kaldmäe

**Affiliations:** Tallinn University, Institute of Mathematics and Natural Sciences, Tallinn, Estonia

Dear Sir,

A recently published article in *The Lancet* (1), focusing on psychiatric disorders and mortality among people in homeless shelters in Denmark, provided through a register-based large-cohort study very interesting, more detailed information and knowledge in this field. 

The Danish data are to some extent in harmony with our own published data from Estonia (2), but there are some discrepancies, e.g. aim, sample size, but also subject recruitment. In particular, the understanding of the term ‘homeless’ can be very different between countries. The question is: what does it mean to be homeless in Denmark where the human development index is very high, compared to some other European countries where the index is high or medium (3)? Through this the physical and mental conditions of homeless people can vary between countries, and also the outcome can differ.

Information about persons living on the streets, who have no identification number and contact with the shelters, is hardly reachable. Also, it is a question about the status ‘homeless’ itself. The study results showed that 44.2% and 61.0% among men and women, respectively, had only one contact with the shelter. The question is if these subjects really were homeless or just persons who had no place to stay for one night. Springer in her article (4) approaches this problem very carefully because every society has different perceptions of such individuals. Still she suggests a global definition, and a person who is staying in a shelter for one night cannot be considered as homeless but more as at risk of homelessness. As a conclusion one way or the other it is quite impossible to get an overview about the real situation of the homeless population. 

Also the study raises a question whether the person was homeless before the contact with the Psychiatric Central or not. There is no doubt about the importance of psychiatric disorders, but according to the literature (including drug abuse) they seem to be a risk factor for homelessness, not an outcome. Folsom et al. (5), in a large-cohort study, show homelessness to be a serious problem among patients with mental illness—not vice versa. Knowing this, it could be a case of a biased sample from the very beginning.

The previous comments do not decrease the value of the study—to reduce psychiatric morbidity and mortality in a homeless group is very important, but still the leading cause of death (considering disease) seems to be cardiovascular diseases (6,7). In conclusion, even with large sample sizes, this kind of population should still be approached methodologically with great care and it would be worthwhile to conduct a study which includes several diseases, not only psychological disorders and substance abuse.
